# Comparison of the accuracy and errors of blood pressure measured by 2 types of non-mercury sphygmomanometers in an epidemiological survey

**DOI:** 10.1097/MD.0000000000010851

**Published:** 2018-06-22

**Authors:** SeongIl Choi, Yu-Mi Kim, Jinho Shin, Young-Hyo Lim, Sung-Yong Choi, Bo-Youl Choi, Kyung-Won Oh, Hyung-Min Lee, Kyung-Ji Woo

**Affiliations:** a Department of Cardiology, Hanyang University Hanmaeum Changwon Hospital, Changwon; b Department of Preventive Medicine, Dong-A University College of Medicine, Busan; c Division of Cardiology, Department of Internal Medicine; d Department of Preventive Medicine, Hanyang University College of Medicine, Seoul; e Division of Health and Nutrition Survey, Korea Centers for Disease Control and Prevention, Osong, Republic of Korea.

**Keywords:** auscultation, blood pressure, diagnostic errors, electronic, epidemiological factors, sphygmomanometers

## Abstract

A few studies have compared auscultation and oscillometric devices with the mercury sphygmomanometer (MS) reference values for blood pressure (BP) measurement in an epidemiologic survey.

Four trained observers recorded BP measurements from 766 subjects from general Korean population in 2014 and 2015. Measurements were repeated 3 times for each device, alternately using an MS and 2 electronic devices (EDs; Greenlight 300 vs Omron HEM-907), together with a randomized device sequence. The BP measurement difference was defined as BP measured by MS minus BP obtained by ED, and the absolute error as the absolute value of the difference.

Mean differences in systolic BP (SBP) were –0.52 and –0.62 mmHg and those of diastolic BP (DBP) were –0.78 and 6.23 mmHg (*P* < .01) in the Greenlight and Omron device group, respectively. The concordance correlation coefficients were 0.97 and 0.94 for SBP and 0.95 and 0.76 for DBP in the Greenlight and Omron group, respectively (*P* < .05). Kappa values for the Joint National Committee 7 BP classification were 0.84 and 0.74 for Greenlight and Omron group, respectively. The prevalence of normotension, prehypertension and hypertension were 53.5%, 33.9%, and 12.5% with the MS and 59.8%, 29.0%, and 11.2% with the ED in the Omron group (*P* = .03, McNemar test), whereas they were insignificant in the Greenlight group.

The Greenlight 300 may be a good alternative to the MS, and the Omron HEM-907 has good accuracy in SBP measurement. Due to the measurement error in DBP, Omron HEM-907 was inferior to the Greenlight device.

## Abstract 2

Supplemental Digital Content is available in the text

## 1 Introduction

Due to the high prevalence and evident health risks of hypertension, blood pressure (BP) measurement is the representative item on the National Health Screening (NHS) Program. Based on robust evidence, a mercury sphygmomanometer (MS) is considered the standard device for BP measurement in epidemiological surveys.^[1,2]^ However, with rapid advances in digital measurement technology, automated BP measuring devices can be successfully used in many real medical practices. Moreover, the environment issue of mercury toxicity can be overcome by replacement of the MS with an electronic digital sphygmomanometer (ED) in the healthcare workplace.^[1–3]^ In the same context, a recent comparative study to replace the MS with an ED was conducted by the National Health and Nutrition Examination Survey (NHANES) Program in the United States. Also, in epidemiological surveys that directly compared EDs with the MS, oscillometric diastolic BP (DBP) and hypertension prevalence were lower for the MS, whereas the non-mercury auscultation BP measurement was similar to that by the MS.^[1,2,4]^ Thus, although the MS currently remains a standard BP measuring device in the NHS program, EDs will ultimately replace the MS in future NHS surveys, because of technological advances and environmental concerns.

Two techniques of non-mercury EDs are currently available to measure BP, including non-mercury auscultation and automated oscillometric. Furthermore, in the presence of a non-uniform measuring device, various types of statistical errors can occur. Hence, the use of a unified BP measuring device is required in large-scale NHS projects. Like the NHANES in the United States, the Korean NHANES is regularly conducted on a large scale and is currently attempting to establish a standard BP measuring device to replace the MS in the future. Although the MS has been replaced with ED in real practice, a few studies have compared an aneroid sphygmomanometer with automated oscillometric BP measurements in population-based studies.

Thus, in the setting of the Korean NHANES, this study compared the BP readings obtained by an MS with those of 2 EDs and ultimately determined which BP measurement device is most suitable to replace MS in future NHS surveys. For this, initial BP measurement differences were obtained between a non-mercury ED, the Greenlight 300 and an MS, and also between the Omron HEM-907 and MS, respectively. Then, the accuracy of the 2 EDs was analyzed through indirect comparison of the differences between the 2 BP measurements. Next, the BP values obtained by the BP measuring devices were categorized by the 2013 Korean Society of Hypertension guidelines for BP classification and the prevalence of hypertension was investigated. Finally, we analyzed the determinants of the BP difference obtained by the 2 BP measuring devices, controlling for parameters.

## 2 Methods

### 2.1 Study participants

Among the subject participating Korean NHANES between May and September 2014 and 2015, we enrolled all consecutive 745 subjects in the four mobile examination units, aged ≥ 20 years, who met the inclusion criteria and who also provided written informed consent. Inclusion criteria included a regular pulse rate during a 15 seconds examination and arm circumference (AC) between 20 and 35 cm. There were no exclusion criteria unless the participant refused to measure BP 6 times. The study protocol was approved by the Institutional Review Board of Hanyang University Hospital.

### 2.2 Before-use and after-use device validation

The Greenlight 300 (Accoson, Essex, United Kingdom) and Omron HEM-907 (Omron, Kyoto, Japan) devices have been validated based on the European Society of Hypertension protocol.^[5,6]^ To ensure the accuracy of the piezoresistive manometer, we performed the validation procedure described in our previous study.^[7]^ In the present study, no difference more than 3 mmHg was observed for the 4 devices applied in the before- and after-use validation tests. Therefore, the previously defined validation criteria were fulfilled.^[8]^ As the inter-device differences were not significant, the devices were not rotated among observers. No device malfunctions were observed.

### 2.3 BP measurements using the mercury sphygmomanometer

Four trained nurses, who collected data for the Korean NHANES, participated in this study to measure BP using the MS (BP_MS_) and ED (BP_ED_). BP measurement protocols adhered to the Korean Center for Disease Control guidelines and were regularly updated to ensure compliance with the American NHANES protocol.^[9]^ Participants were seated in a chair with back support, in a quiet room, with both feet resting comfortably on the floor and right arm, where the BP measurements were taken, supported on a level surface with the cubital fossa at heart level. The first and the fifth Korotkoff sounds were recorded for the systolic BP (SBP) and DBP, respectively. The selection of cuff size, arm level, deflation speed, and other quality control issues were described in a previous study.^[7]^ The standard quality control protocol was carried out and fulfilled by passing the tests, and the BP measurement difference was ≤ 2 mmHg for the SBP and DBP, respectively. BP was measured at least 30 seconds apart, following a minimum 5 minutes rest.

### 2.4 BP measurements using electronic device

The MS and EDs were alternately used to record triplicate BP measurements per patient, with the same appropriate-sized cuffs, the same arm, and the same posture. The order of BP measurement and the observers were randomly assigned to either the MS or the EDs (Greenlight 300 vs Omron HEM-907) to reduce measurement bias. With the Greenlight device, it was not possible to control memory bias with the automatic mechanical method. To monitor memory bias and to demonstrate the validity of the BP measurement, the group using the Omron was divided into Omron I (the Omron was used first) and Omron II (the MS was used first) (see Figure, Supplemental Content 1, which illustrates the study flow chart).

Also, to facilitate post-hoc comparison and interpretation, the observer was not blinded to the Omron reading. The selection of cuff size was based on the manufacturer's guidelines for each device. For the BP measurement using the Greenlight device, SBP was determined by the first Korotkoff sound and DBP by the fifth Korotkoff sound, rounded to the nearest 2 mmHg. All BP measurements were repeated at 30 seconds intervals, following a minimum 5 minutes rest.

### 2.5 Definitions of measurement differences and errors

Based on the recommended technique for measuring BP from the Canadian Hypertension Education Program^[10]^ and Korean NHANES protocol, the first reading was discarded, and the average value of the last 2 measurements was used for the analysis. Thus, the SBP measurement difference (d-SBP) between the MS and ED was defined as the average of the second and third SBP measurements taken with the MS (SBP_MS_) minus the average of the second and third SBP measurements taken with the ED (SBP_ED_). The DBP measurement difference (d-DBP) was defined as the average of the second and third DBP measurements taken with the MS (DBP_MS_) minus the average of the second and third DBP measurements taken with the ED (DBP_ED_). Absolute error was defined as the absolute value of the difference in SBP (a-SBP) and DBP (a-DBP).

### 2.6 Comparison of BP classification

Based on the 2013 Korean Society of Hypertension guidelines,^[11]^ BP classification was categorized into normotension, prehypertension, and hypertension. Hypertension and prehypertension were not divided into stage I and II. Hypertension was defined based only on BP level, without information regarding antihypertensive medication status.

### 2.7 Statistical analyses

The sample size was calculated based on a power of 0.9 and an alpha of 0.05. Comparisons of the MS to the ED were performed using Lin's concordance correlation coefficients (CCC). We assumed the minimum threshold of CCC should be 0.90 and H0 = 0.90 and H1 = 0.925. With type I error of 0.05 for 2-tailed test and study power of 0.9, sample size is 380.^[12]^ Data are presented as the mean ± standard deviation, number (%) or median. General participant characteristics were described according to the gender and the assigned groups to the 2 EDs. Characteristics between sex and the groups were compared using student *t*-test and *χ*^*2*^ test without adjustment. As the difference between the MS and ED showed a non-Gaussian distribution, data are displayed using the median and interquartile range. Median values were compared using Friedman 2-way nonparametric analysis of variance. Pearson correlation coefficient was calculated, and a Bland-Altman plot was created. Multiple linear regression models were constructed to identify factors independently associated with BP measurement errors. Model 1 included SBP_ED_ or DBP_ED_, age, gender, height, and AC. Given that AC, body weight and body mass index showed colinearity, only AC was included in the model. Model 2 additionally included the sequence of measurements, with ‘MS first’ used as the reference. For the analysis of the impact of age and AC on the difference and absolute error, the least square means adjusted for quartiles (Q) of age and AC, as well as for gender, were tested. An adjusted kappa value was calculated to determine the reliability of the BP classification. For differences between the BP classification and the prevalence and control rate of hypertension, McNemar *χ*^*2*^ test was performed. Statistical analysis was achieved using the Statistical Analysis System software package version 9.2 (SAS Institute Inc., Cary, NC). All *P *<.05 were regarded as statistically significant. For indirect comparison, the 95% confidence interval (CI) was used to determine statistical significance.

## 3 Results

### 3.1 General participant characteristics

A total of 766 participants were recruited and 447 subjects (58.3%) were female. Age was similar between sexes (51.8 ± 17.5 vs 52.1 ± 16.2 years in males and females respectively, *P* = .80). Body mass index differed between sexes (23.9 ± 2.9 vs 23.4 ± 3.2 kg/m^2^, respectively, *P* = .02), as did AC (28.1 ± 2.2 vs 26.6 ± 2.3 cm, respectively, *P* < .01).

### 3.2 Comparison of BP_MS_ and BP_ED_

#### 3.2.1 Baseline characteristics

Baseline characteristics were similar between the Greenlight and the Omron group (Table 1), (see Table, Supplemental Content 2, which shows general characteristics of the study subject). Mean SBPs obtained by the MS and EDs were similar. In contrast, the mean DBPs were markedly different between the ED types and those recorded by the MS. Specifically, d-SBP was similar between both groups, and the values fell well within the permissible range difference defined by the Association for the Advancement of Medical Instrumentation.^[13]^ However, d-DBP values were significantly larger in the Omron group than the Greenlight group, and the differences were outside the permissible range. The Omron group had higher a-SBP values and much higher a-DBPs than the Greenlight cohort. Thus, compared to BP values obtained by the MS, the Omron group showed similar SBP values but significantly lower DBPs.

**Table 1:**
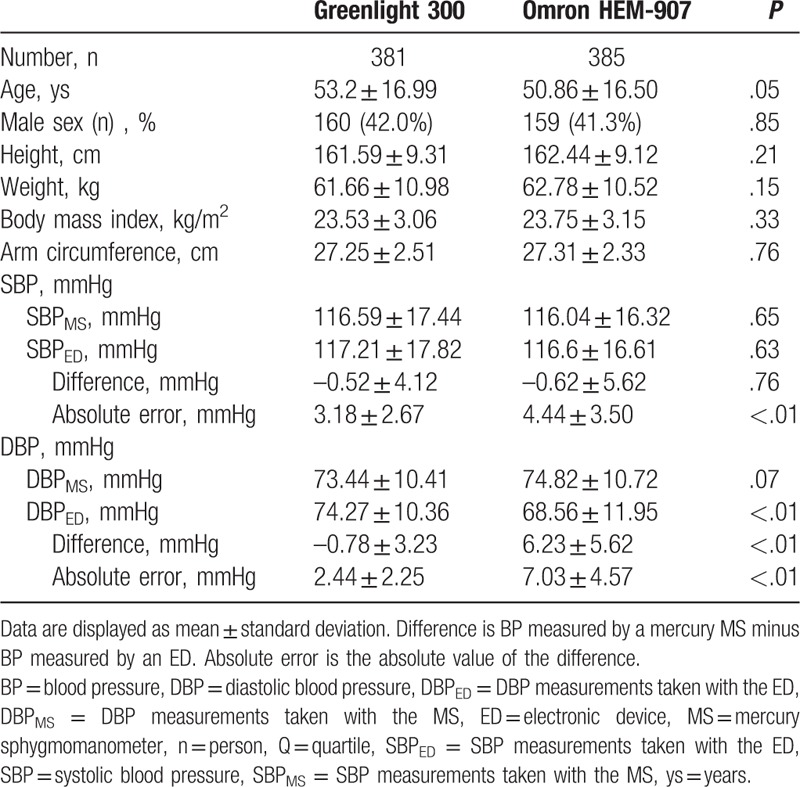
General characteristics of the study subject.

#### 3.2.2 Correlation of BP_MS_ and BP_ED_

**Figure 1:**
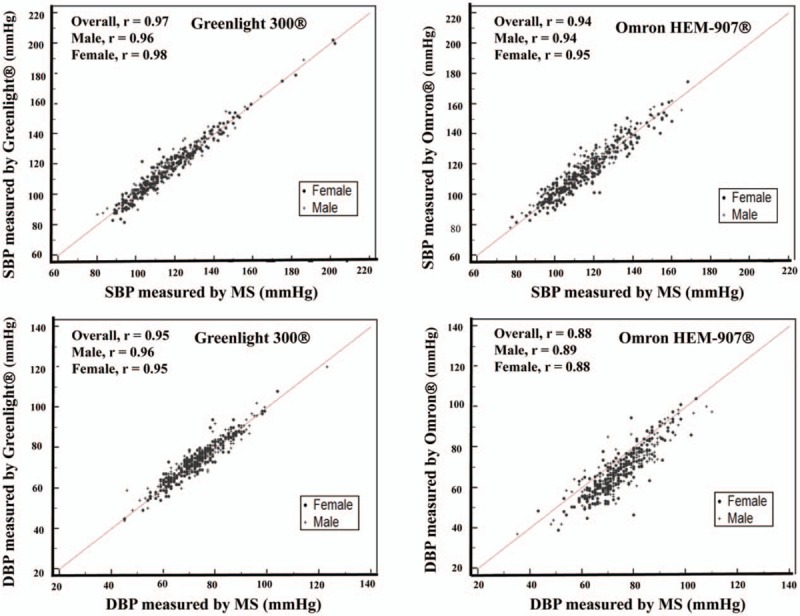
Correlation between BP measurements recorded with a MS and Greenlight 300 versus MS and Omron HEM-907. Pearson correlation coefficients for systolic BP were comparable between Greenlight 300 and Omron HEM-907 (upper panels). However, Pearson's correlation coefficient for diastolic BP, Omron HEM-907 was inferior to Greenlight 300 (lower panels). BP = blood pressure, DBP = diastolic blood pressure, ED = electronic device, Greenlight = Greenlight 300, MS = mercury sphygmomanometer, Omron = Omron HEM-907, SBP = systolic blood pressure.

As shown in Figure 1, the correlation coefficients (R^2^) for SBP were very high in both ED groups (R^2^ = 0.95 and 0.89 for Greenlight and Omron group, respectively), whereas for DBP, the correlation coefficient in the Omron group was a little lower (R^2^ = 0.90 and 0.78, respectively). The CCCs in Table 2 showed similar results. The lower margin of 95% CI of CCCs in SBP and DBP were higher than 0.85 for Greenlight group. However, in Omron group, the lower margin of 95% CI of CCC was higher than 0.85 only in SBP, but CCC for DBP was not acceptable suggesting poor strength of agreement. That is, the CCCs were significantly higher for both SBP and DBP in the Greenlight cohort than the Omron group. Furthermore, the CCCs were affected by age and AC in the Omron group, showing significant differences between Q1 and Q4.

**Table 2:**
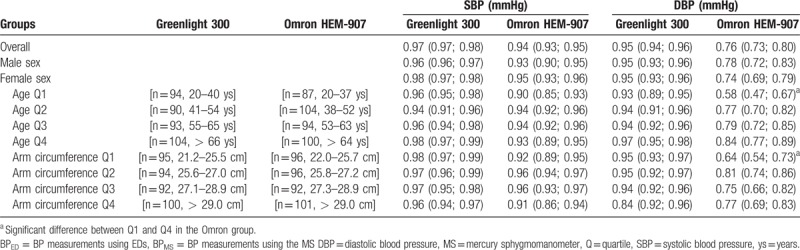
Concordance correlation coefficients between BP_MS_and BP_ED_.

d-SBP and d-DBP were higher and scattered widely in the Omron group, where females exhibited a proportional correlation with d-DBP (r = –0.34, *P* < .01) (Fig. 2).

**Figure 2:**
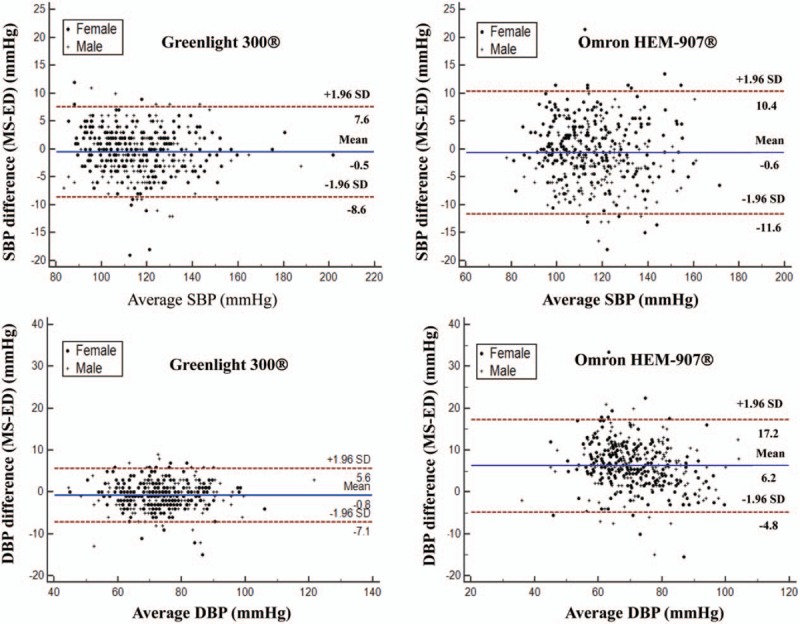
Bland-Altman plots comparing the difference in BP measurements obtained with a MS and Greenlight 300 versus MS and Omron HEM-907. Left and right panels for Greenlight 300 and Omron HEM-907, respectively. The mean of the difference in the SBP (d-SBP) was similar between the 2 devices, but the difference was distributed much more widely with the Omron HEM-907 than the Greenlight 300 (upper panels). Compared to the Greenlight 300 (lower panels), a higher mean difference in the DBP (d-DBP) and markedly wider distribution of the difference was obtained with the Omron HEM-907. a-SBP = absolute value of difference in SBP, BP = blood pressure, DBP = diastolic blood pressure, ED = electronic device, Greenlight = Greenlight 300, MS = mercury sphygmomanometer, Omron = Omron HEM-907, SBP = systolic blood pressure, SD = standard deviation.

**Figure 3:**
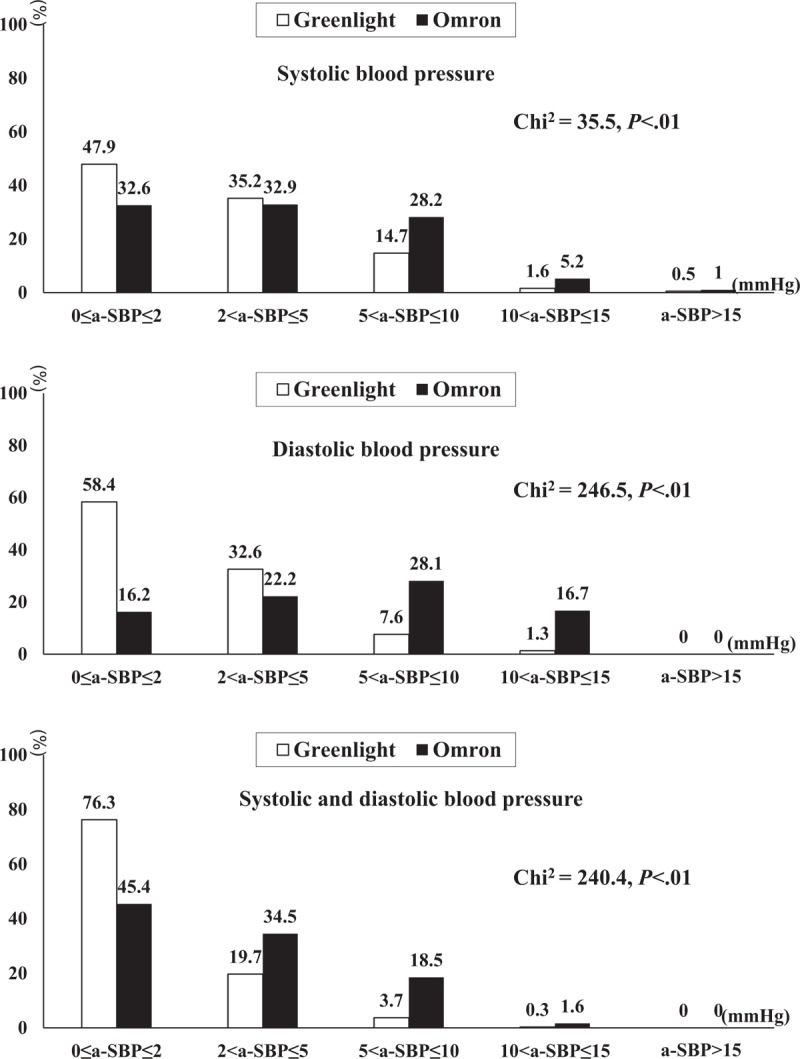
Distribution of absolute error. The distribution of the absolute error was defined as the absolute value of the differences in BP between a MS and Greenlight 300 versus MS and Omron HEM-907. Absolute errors of SBP by Greenlight 300 were distributed towards the smaller ranges than the Omron HEM-907 (upper panel). The distribution pattern of the absolute error of DBP was similar and more evident (middle panel). Combined distribution of the absolute error of SBPs and DBPs was much more different in the range of absolute error higher than 5 mmHg (lower panel). BP = blood pressure, DBP = diastolic blood pressure, MS = mercury sphygmomanometer, SBP = systolic blood pressure.

As shown in Figure 3, the absolute error was distributed in a significantly different pattern between the Greenlight and Omron groups. An absolute error in SBP or DBP > 10 mmHg was present in 22.9% of the Omron group and such difference was mainly attributable to a-DBP (*χ*^*2*^ = 246.5, *P* < .01). In contrast, 96% of subjects in the Greenlight cohort had an absolute error in both SBP and DBP of < 5 mmHg.

#### 3.2.3 Between-device agreement by the Joint National Committee 7 BP classification

As shown in Table 3, the prevalence of normotension, prehypertension, and hypertension was respectively 52.9%, 35.0%, and 12.1% for the MS and 50.3%, 36.6%, and 13.1% for the Greenlight ED (*P* = .21). The corresponding values were 59.8%, 29.0%, and 11.2% for the Omron ED and 53.5%, 33.9%, and 12.5% for the MS. Thus, the prevalence of hypertension classified by the Greenlight 300 was 1% higher than that of hypertension defined by the MS, whereas the prevalence of hypertension by the Omron device had a 1.3% lower incidence compared to the hypertension defined by the MS. These significant differences were attributed to the lower DBP effect and occurred mainly in the discrepancy between prehypertension and normotension (*P* = .03). The adjusted kappa for BP classification was 0.84 and 0.74 for the Greenlight and Omron group, respectively. Thus, although the agreements in the BP classification of normotension, prehypertension, and hypertension by the 2 EDs were above those to be expected by chance, the agreement was lower for the Omron than the Greenlight device.

**Table 3:**
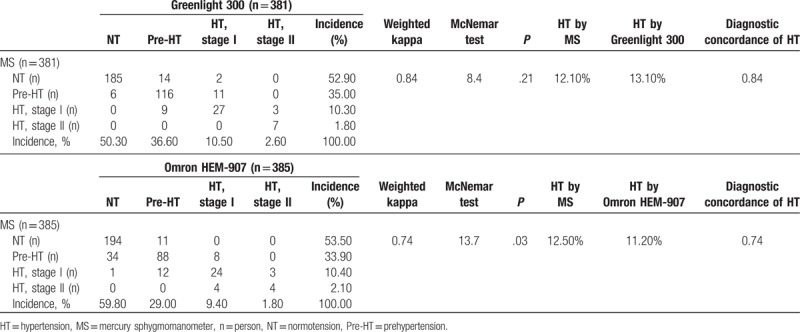
Diagnostic agreement and concordance of hypertension.

#### 3.2.4 Risk factors for differences and absolute errors

The BP differences measured using the MS (BP_MS_) and ED (BP_ED_) were affected by age, AC, and the type of BP measuring device (ED type). As shown in Table 4, BP_ED_ and AC were common determinants for difference between BP_MS_ and BP_ED_ in the Greenlight cohort, whereas in the Omron cohort, age and BP_ED_ were significant effectors for d-DBP, but d-SBP was associated with AC and female. In model 2 in Table 4, the measurement sequence of Omron was a significant factor for d-SBP. However, the beta value was only –1.31 mmHg, indicating that the memory bias between both groups may be acceptable.

**Table 4:**
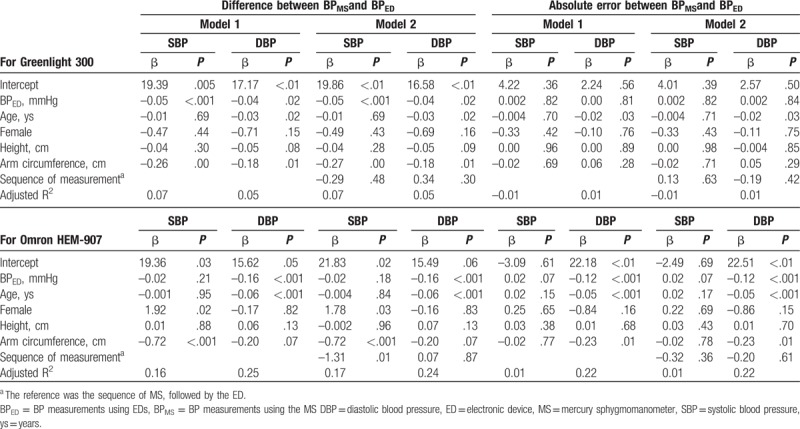
Factors determining difference and absolute error in BP_MS_ and BP_ED_ in the multiple linear regression models.

Unlike the BP difference between BP_MS_ and BP_ED,_ absolute errors between BP_MS_ and BP_ED_ varied by BP types (SBP/DBP). As shown in Table 4, no parameter was a significant effector for a-SBP. Meanwhile, age was a common determinant for a-DBP, and ED type and AC were also significant factors for a-DBP in the Omron cohort. However, regardless of predictive model types, the adjusted R^2^ values for Greenlight or Omron were overall low (R^2^ = –0.01 to 0.25).

#### 3.2.5 Differences according to the arm circumference and age

As shown in Table 5, d-SBP varied among age quartile groups with Greenlight, whereas d-DBP varied by age with Omron, and was the lowest in the oldest quartile (Q4; > 64 years). Meanwhile, AC quartiles were inversely related to d-SBPs and d-DBPs in both groups (all *P* for trend <.01). Therefore, irrespective of ED type, d-SBPs and d-DBPs were the smallest in subjects with the largest AC quartile (Q4; > 29 cm).

**Table 5:**
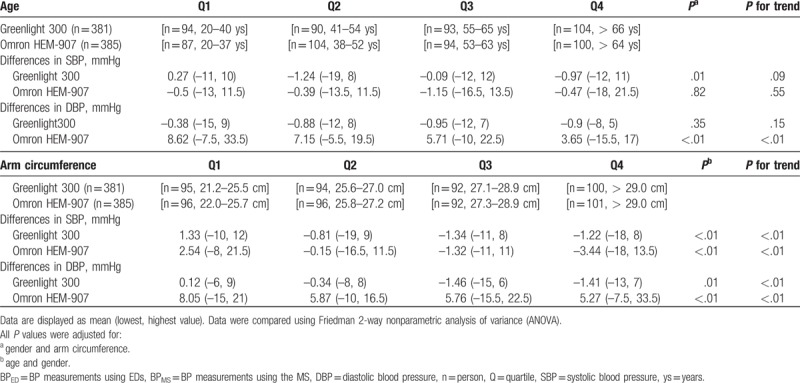
Differences between BP_MS_ and BP_ED_ according to age and arm circumference quartiles.

As shown in Table 6, a-SBP was not affected by age or AC in any of the groups, whereas age or AC affected a-DBP in the Omron group, and showed a negative linear trend for all four AC and age groups (all *P* for trend <.01). In summary, d-DBP and a-DBP were relatively smaller in older subjects and subjects with a larger AC.

**Table 6:**
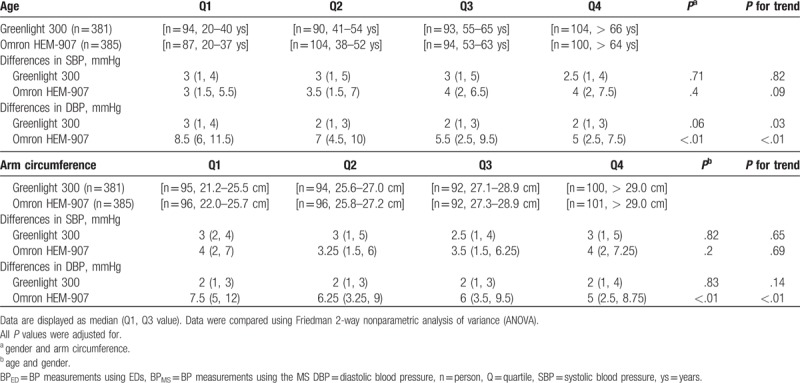
Absolute error between BP_MS_ and BP_ED_ according to age and arm circumference quartiles.

## 4 Discussion

This study investigated the BP measuring devices that can replace MS in future epidemiology surveys (ie, Korean NHANES) and the impact of replacing a BP measurement device on the interpretation of epidemiological data. The main findings were that the Greenlight and MS recorded comparable BP measurements, whereas SBP measured by the Omron was similar to the MS data, but DBP was lower for the Omron. Also, the prevalence of hypertension was also lower. Namely, the accuracy of SBP measurement was comparable, while the CCC for DBP measurement was significantly lower in the Omron group than the Greenlight group. The absolute error and its SD, in this study, were comparable to the intrinsic variability of BP shown in a study to evaluate inter-arm differences.^[14]^

Despite the opposite result in the 2011 British survey,^[15]^ our result is similar to several previous clinical results and the result shown in the NHANES survey environment.^[1,2,4,16]^ However, surprisingly, MS is currently replaced by the Omron devices (ie, oscillometric method) in most clinical situations. According to the 2011 study published in the United Kingdom,^[15]^ the ratio of BP measurement by using ED: MS was 4:1, which is ascribed to the advantages of automatic repeated measurement in the oscillometric method. Particularly, in the emergency room, intensive care unit or operating room, the automatic repeated measurement of the Omron is useful for monitoring the patient's condition rather than the confirmatory diagnosis of hypertension and also compensates for the measurement error. In contrast, the precise measurement is most important in nationwide NHS surveys because an accurate census should be obtained from a single BP measurement. Moreover, considering the continuity with previous data or the existing statistics, BP measurement errors may distort the trend in prevalence of hypertension and control rate.

In our study, the Greenlight device showed a 1% higher prevalence of hypertension than MS and Omron had a 1.3% lower prevalence of hypertension. This between-device agreement for the frequency of hypertension was greater than that reported in the previous NHANES survey, where, compared to the MS, the Greenlight and Omron devices underestimated the prevalence of hypertension by 1.66% and 2.65%, respectively.^[1,2]^

Our result demonstrated that the Greenlight device achieved accuracy comparable to the MS in an epidemiological survey setting. Hence, EDs like Greenlight 300 that employ auscultation methods can replace the conventional MS within the acceptable range of approved measurement errors.^[2]^ However, as the Greenlight device has issues with long-term reliability and observer error,^[17]^ the accuracy of BP measured by this device is guaranteed only when a proper maintenance protocol is followed.^[3]^

On the contrary, the Omron device cannot fully replace the MS. Our study finding that DBP measured by the Omron was on average 6 mmHg lower than DBP_MS_ is consistent with the results of the original validation studies for the Omron 907, in which the mean DBP was about 4 to 5 mmHg lower than DBP_MS._^[2,18]^ It means that diastolic hypertension may be diagnosed as below 90 mmHg when an Omron device is used to measure the DBP. Moreover, given that lowering the DBP by 5 mmHg reduces the risk of stroke by an estimated 34% and ischemic heart disease by 21% from any pre-treatment level,^[19]^ an underestimation of DBP by an average of 4 to 6 mmHg may result in greater risks of erroneous decisions. This finding emphasizes that a different hypertension cut-off level should be used to correctly diagnose hypertension when the BP reading is obtained by an Omron. In this study, the absolute error distribution measured by Omron HEM-907 was similar to data from the NHANES participants.^[2]^ Thus, based on the significant measurement differences between the MS and Omron devices, the Omron device should not be considered interchangeable with the MS.^[20]^

At an individual level, the measurement errors discussed above may eventually lead to an inappropriate diagnosis of hypertension and under-treatment in hypertension, whereas regarding the national public health; it hinders the early detection of hypertension and prevention of complications. Thus, BP measurement using the manual auscultation is a major disadvantage, compared to an automated oscillometric method. However, the MS should be preferably replaced with a non-mercury aneroid auscultation method (ie, Greenlight 300), in epidemiological surveys, for accurate BP measurement and statistical reliability. Moreover, the auscultation technique cannot be circumvented, and the misconception that abandoning the mercury technique means relinquishing auscultation altogether should be avoided.

A notable feature of this study is the indirect comparison of the ED and the MS. In prior studies or validation tests, the MS and ED were, individually and directly, compared with a 1:1 matching.^[1,2,4]^ Meanwhile, in this study, the MS and Omron were compared directly within one group, and a direct comparison of MS and Greenlight in the other group. Finally, BP differences shown within individual groups were indirectly compared between both groups. For a direct comparison of all devices (Greenlight and Omron) with the gold standard (MS), triplicate measurements are needed per device, together with a randomized device sequence. However, 9 repeated BP measurements per subject are not feasible in real-world situations, and BP variability or serial changes could limit the applicability or generalizability of the study results. Also, oscillometric devices are not subject to memory bias. Furthermore, a minimum 30 seconds interval between BP measurements interval must also be maintained, and the 9 repeated BP measurements may eventually augment the white-coat effect. Hence, despite similar baseline characteristics between both groups, this is controversial. However, this study design reflects the real practice anticipated in future epidemiological surveys. Our study intended to assess the accuracy of the ED models in an epidemiological setting and focused solely on how to apply the previously documented data in the nationwide NHS program. Previous studies have used 6 sequential measurements, alternating between the reference (MS) and the test device. Moreover, although the validation does not guarantee the accuracy of all measuring instruments, Greenlight and Omron devices have been validated and widely used. Thus, we did not exactly follow the validation protocol set by the Association for the Advancement of Medical Instrumentation or British Hypertension Society. As a result, this study design may be useful to compare at least 3 BP measuring devices.

Finally, our study found that the common errors of BP_MS_ were associated with age, AC and the type of ED measuring device, in concurrence with several previous studies.^[2,3,21]^ Expectedly, age was negatively correlated with the between-device differences.^[2]^ Namely, the differences between BP_MS_ and BP_ED_ were not significant in the elderly (aged > 65 years), whereas those aged < 40 years had significant differences between BP_MS_ and BP_ED_. Previous studies also showed a significant difference between device readings for youth.^[2,20]^ Moreover, the slightly larger BP measurement differences reported in this study compared to literature data may be ascribed to an age composition difference that identified exaggerated differences in the younger subjects (aged < 40 years). This finding supports the recommendation of using the Omron more often with the elderly (aged > 65 years). Like age, AC was also negatively correlated with the between-device differences, which were greater in the smallest AC group, relative to the largest AC group. Thus, the more significant differences were observed in younger subjects and subjects with a smaller AC.

Meanwhile, in our study, the impact of BP and lean body mass on the differences between devices contrasted with the literature results.^[2,4,21]^ Previous studies consistently showed that both increased SBP and DBP, decreased device agreement between the Omron and MS.^[2,4]^ However, in our study, Bland-Altman plots did not show any trends towards greater differences between BP_MS_ and BP_ED_ at higher or lower BP values. Instead, female patients were underestimated for DBP measurements below 70 mmHg compared to the male patients. This finding might be attributed to the general, smaller AC in women.

## 5 Summary

This study compared BP measurements from 2 EDs (Greenlight 300 and Omron HEM-907) and the MS in an epidemiological survey setting. Both EDs showed excellent performance for measuring SBP. However, for measuring DBP, the Greenlight 300 exhibited significantly better results than the Omron HEM-907. For BP classification, Omron HEM-907 performance was inferior to the MS due to misclassification results for a non-hypertension population. Hence, although there is no perfect method to replace BP measurements by the MS, the Greenlight 300 can be considered a more reasonable alternative to the MS for BP measurement than the Omron device, in an epidemiological survey focused on the general population, particularly, including younger and normotensive subjects.

## 6 Limitations

This study is fundamentally an indirect or post-hoc comparison study. Although the study situation was similar and the randomized allocation method was performed in specific sequences to compare the Greenlight and Omron devices, limitations were identified, because, for direct comparison, triple triplicates of BP readings are essential. Particularly, our study is limited to an interpretation of the direct comparison between 2 devices. A further investigation, with a better study design, is needed to draw more solid conclusions. Second, as only 1 instrument of each type was tested in this study, it is impossible to conclude the overall effectiveness of aneroid and digital instruments. Also, the result cannot be generalized to all individuals.

## Author contributions

All authors contributed to the writing or revision of the article. All authors had full access to all of the study data and take responsibility for the integrity of the data and the accuracy of data analysis. All authors read and approved the final article.

**Data analysis:** Young-Hyo Lim, Sung-Yong Choi, Bo-Youl Choi, Yu-Mi Kim.

**Data curation:** Kyung-Won Oh, Hyung-Min Lee, Kyung-Ji Woo.

**Design:** Jinho Shin.

**Formal analysis:** Jinho Shin, Sung-Yong Choi, Yu-Mi Kim.

**Funding acquisition:** Jinho Shin.

**Investigation:** Jinho Shin.

**Methodology:** Jinho Shin.

**Participant recruitment:** Kyung-Won Oh, Hyung-Min Lee, Kyung-Ji Woo.

**Project administration:** Jinho Shin.

**Validation:** Sung-Yong Choi, Bo-Youl Choi

**Writing – original draft:** SeongIl Choi, Jinho Shin.

**Writing – review & editing:** SeongIl Choi, Yu-Mi Kim.

## Supplementary Material

Supplemental Digital Content
