# Supplementary material for: Comparison of the accuracy and errors of blood pressure measured by 2 types of non-mercury sphygmomanometers in an epidemiological survey
Source: Medicine (Baltimore). 2018 Jun 22;97(25):e10851. doi: 10.1097/MD.0000000000010851 (PMC6023853; doi:10.1097/MD.0000000000010851)
Supplement: Supplemental Digital Content [file medi-97-e10851-s001.docx]

Supplemental Digital Content 1. Figure that illustrates the study flow chart.


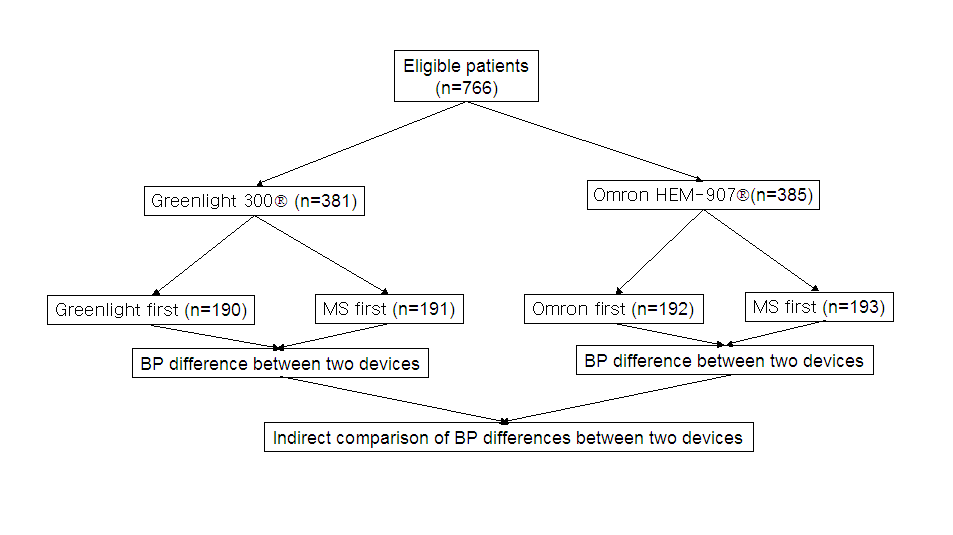


| **Supplement 2.** General characteristics of the study subject | | | | | |
| --- | --- | --- | --- | --- | --- |
|  | Greenlight 300® | Omron HEM-907® | Greenlight 300® | Omron HEM-907® | *P* value |
| Number (*n*) | 381 | 385 | 381 | 385 |  |
| Systolic BP (mmHg) |  |  |  |  |  |
| by MS (mmHg) |  |  | 116.59±17.44 | 116.04±16.32 | 0.65 |
| Age Q1 (mmHg) | [*n* = 94, 20-40 years] | [*n* = 87, 20-37 years] | 108.26±14.18 | 108.24±11.16 | 0.99 |
| Age Q2 (mmHg) | [*n* = 90, 41-54 years] | [*n* = 104, 38-52 years] | 111.37±13.71 | 112.19±15.31 | 0.70 |
| Age Q3 (mmHg) | [*n* = 93, 55-65 years] | [*n* = 94, 53-63 years] | 119.18±15.16 | 121.3±17.22 | 0.37 |
| Age Q4 (mmHg) | [*n* = 104, > 66 years] | [*n* = 100, > 64 years] | 126.31±19.59 | 121.88±16.51 | 0.08 |
| Arm circumference Q1 (mmHg) | [*n* = 95, 21.2-25.5 cm] | [*n* = 96, 22.0-25.7 cm] | 114.81±20.29 | 111.55±15.55 | 0.22 |
| Arm circumference Q2 (mmHg) | [*n* = 94, 25.6-27.0 cm] | [*n* = 96, 25.8-27.2 cm] | 116.31±18.12 | 115.73±17.17 | 0.82 |
| Arm circumference Q3 (mmHg) | [*n* = 92, 27.1-28.9 cm] | [*n* = 92, 27.3-28.9 cm] | 118.98±15.63 | 116.11±17.00 | 0.24 |
| Arm circumference Q4 (mmHg) | [*n* = 100, > 29.0 cm] | [*n* = 101, > 29.0 cm] | 116.33±15.33 | 120.53±14.48 | 0.05 |
| by ED (mmHg) |  |  | 117.21±17.82 | 116.6±16.61 | 0.63 |
| Difference (mmHg) |  |  | -0.52±4.12 | -0.62±5.62 | 0.76 |
| Absolute error (mmHg) |  |  | 3.18±2.67 | 4.44±3.50 | <0.01 |
| Diastolic BP (mmHg) |  |  |  |  |  |
| by MS (mmHg) |  |  | 73.44±10.41 | 74.82±10.72 | 0.07 |
| Age Q1 (mmHg) | [*n* = 94, 20-40 years] | [*n* = 87, 20-37 years] | 73.52±10.39 | 72.89±9.31 | 0.67 |
| Age Q2 (mmHg) | [*n* = 90, 41-54 years] | [*n* = 104, 38-52 years] | 75.64±9.66 | 75.5±10.73 | 0.93 |
| Age Q3 (mmHg) | [*n* = 93, 55-65 years] | [*n* = 94, 53-63 years] | 75.65±9.57 | 79.09±10.32 | 0.02 |
| Age Q4 (mmHg) | [*n* = 104, > 66 years] | [*n* = 100, > 64 years] | 69.49±10.74 | 71.78±10.96 | 0.13 |
| Arm circumference Q1 (mmHg) | [*n* = 95, 21.2-25.5 cm] | [*n* = 96, 22.0-25.7 cm] | 70.88±9.24 | 71.58±10.18 | 0.62 |
| Arm circumference Q2 (mmHg) | [*n* = 94, 25.6-27.0 cm] | [*n* = 96, 25.8-27.2 cm] | 72.79±9.92 | 73.92±10.79 | 0.45 |
| Arm circumference Q3 (mmHg) | [*n* = 92, 27.1-28.9 cm] | [*n* = 92, 27.3-28.9 cm] | 73.36±10.09 | 73.66±10.07 | 0.84 |
| Arm circumference Q4 (mmHg) | [*n* = 100, > 29.0 cm] | [*n* = 101, > 29.0 cm] | 76.56±11.50 | 79.82±10.12 | 0.03 |
| by ED (mmHg) |  |  | 74.27±10.36 | 68.56±11.95 | <0.01 |
| Difference (mmHg) |  |  | -0.78±3.23 | 6.23±5.62 | <0.01 |
| Absolute error (mmHg) |  |  | 2.44±2.25 | 7.03±4.57 | <0.01 |
| Data are displayed as mean ± standard deviation. Difference is blood pressure (BP) measured by a mercury sphygmomanometer (MS) minus BP measured by an electronic device (ED). Absolute error is the absolute value of the difference.  Abbreviations: BP, blood pressure; ED, electronic device; MS, mercury sphygmomanometer; Q, quartile. | | | | | |
